# Risk Factors of Mortality in Patients With Periprosthetic Fractures: An Experience of 100 Cases

**DOI:** 10.7759/cureus.79863

**Published:** 2025-02-28

**Authors:** Irfan Ahmad, Junaid Zeb, Haider Ayyaz, Hafiz Salman Mushtaq, Mariam Aziz, Fouad Chaudhry

**Affiliations:** 1 Orthopaedics, Royal Alexandra Hospital, Paisley, GBR; 2 Trauma and Orthopaedics, Heartlands Hospital, Birmingham, GBR; 3 Trauma and Orthopaedics, Russells Hall Hospital, Dudley, GBR; 4 Trauma and Orthopaedics, Royal Derby Hospital, Derby, GBR; 5 Anaesthesiology, King Edward Medical University (KEMU), Lahore, PAK

**Keywords:** hip replacement, knee replacement, mortality, nonoperative management, operative management, periprosthetic fractures, risk factors, vancouver classification

## Abstract

Objective: This study aims to determine the frequency and risk factors of mortality in patients with periprosthetic fractures.

Materials and methods: A retrospective review was conducted on 100 patients with periprosthetic fractures around hip and knee replacements. Inclusion criteria were radiological evidence of fracture and age >65 years. Exclusions included prior surgically treated fractures, fractures due to malignancy, lost follow-up, or unavailable postoperative data. Patients were analyzed for postoperative fracture union, complications, and mobility status using clinical and radiographic data. Data were analyzed using R software version 4.3.3 (R Foundation for Statistical Computing, Vienna, Austria), employing chi-square and Mann-Whitney U tests for categorical and numerical data, respectively, with logistic regression to control for confounders.

Results: The mean time to operation was 3.25 ± 1.11 days. Among 100 patients, the mean age was 82.22 ± 6.90 years, with a slight male predominance n = 58 (58%). Hip fractures were more common (n = 73; 73%), with knee fractures comprising n = 27 (27%). Most patients were treated at district general hospitals (DGHs) (n = 86; 86%). Mortality within six months was n = 17 (17%). No significant gender differences in mortality were found (p = 0.3). Age >81 years was associated with higher mortality (p = 0.04). Nonoperative management was linked to higher mortality (p = 0.003). No significant differences were observed between hip and knee fracture mortality rates (p = 0.8) or across fracture complexity (p = 0.5). Multivariate analysis indicated higher mortality for nonoperated patients (OR: 0.18, p = 0.004).

Conclusion: Age and operative management significantly impacted mortality in periprosthetic fractures. Older age (≥81 years) and nonoperative management were linked to higher mortality rates.

## Introduction

Periprosthetic fractures, which occur around joint prostheses, are a serious and increasingly prevalent complication in orthopedic practice [1], with an incidence ranging from 0.1% to 4.1% [2]. These fractures pose significant clinical challenges due to their complexity and the vulnerable nature of the patient population, which often includes elderly individuals with multiple comorbidities [3]. With the global increase in joint replacement surgeries, particularly among the aging population, the incidence of periprosthetic fractures is expected to rise [4]. These fractures are associated with high rates of morbidity and mortality, making them a critical area of concern in orthopedic care [5].

Despite advancements in surgical techniques, prosthetic materials, and postoperative management, the outcomes for patients with periprosthetic fractures remain concerning. Mortality rates following these fractures are notably high, reflecting the severity of the injury and the frailty of the affected patients [6]. Previous studies have highlighted various factors that may influence mortality, including the patient’s age, gender, overall health status, the type and location of the fracture, the complexity of the fracture according to classifications such as the Vancouver classification, and the quality and setting of the medical care provided.

Understanding the frequency and determinants of mortality in patients with periprosthetic fractures is crucial for several reasons. Firstly, it enables healthcare providers to better assess and manage the risks associated with these fractures. Secondly, it can inform clinical decision-making, helping to tailor treatment approaches to individual patient profiles and thus improve outcomes. Thirdly, identifying modifiable risk factors can lead to the development of targeted interventions and preventative strategies, ultimately reducing mortality rates in this vulnerable patient group.

This study aims to provide a comprehensive analysis of the frequency of mortality in patients with periprosthetic fractures and to identify the key factors associated with increased mortality risk. By examining a range of patient demographics, fracture characteristics, and treatment variables, we seek to elucidate the multifaceted nature of these fractures and their outcomes. Our goal is to enhance the understanding of periprosthetic fracture management and to contribute to the development of strategies that can improve survival rates and quality of care for these patients. The objective of this study was to determine the frequency and risk factors for mortality in patients with periprosthetic fractures.

## Materials and methods

Study design: retrospective

Study Population

All the patients who presented with periprosthetic fractures around hip and knee replacement from January 2017 to December 2022 who were admitted under Trauma and Orthopaedics at Russells Hall hospital, The Dudley Group NHS Foundation Trust, were retrospectively reviewed using the hospital database.

Inclusion: Patients were included if they had radiological evidence of fracture and were >65 years old. Patients were excluded if they had a prior fracture that was surgically treated, they had fractures due to malignancy, they were lost at follow-up, or their postoperative radiographs or data were not available for review. During this period, around 139 patients presented with periprosthetic fractures around the hip and knee, and 100 patients who fulfilled the inclusion criteria were analyzed.

Sample size: A total of 100 patients were recruited into the study, those with periprosthetic hip fractures. The fractures were classified using the Vancouver classification by two orthopedic surgeons. Fractures were categorized into types A, B, and C. Type B fractures were further subclassified into B1, B2, and B3. Additionally, the sample included patients with periprosthetic fractures around total knee replacements. Patients were further subdivided based on treatment into three categories: those who were managed nonoperatively with non-weight-bearing mobilization protocol for six weeks and gradually progressing to partial and full weight-bearing, those managed operatively, and those who were managed with referral to designated specialized center for surgical management. All patients with Vancouver A were managed nonoperatively, and all the fractures united without any complication. Out of 29 Vancouver B1 fractures, 26 were managed with surgical open reduction and internal fixation with NCB Zimmer plates and cables, while three patients were managed conservatively as they were unable to tolerate surgery. Out of 26 operated patients, 24 were operated in the local district general hospital (DGH), and two were operated in the tertiary care hospital. All 16 B2 fractures were managed with surgical options; 10 were managed with open reduction and internal fixation with NCB Zimmer plates and cables in the local DGH hospital; and six patients had revision of the femoral stem. Out of these six revision surgeries, four were done in the local DGH, and two were performed in the tertiary care hospital. Out of 12 Vancouver B3 fractures, 10 patients underwent revision arthroplasty surgery, and all revision surgeries were performed in the tertiary care hospital. The remaining two patients were unable to undergo surgical intervention due to surgical fitness issues and were managed conservatively in the local DGH hospital after discussion with the tertiary care hospital. All eight Vancouver C fractures were managed with open reduction and internal fixation with NCB Zimmer plates and cables at the local DGH hospital. With regard to the 27 periprosthetic fractures around the knee, 10 fractures were managed conservatively due to acceptable implant position and undisplaced fractures. Of the remaining 17 fractures, 11 were managed with open reduction and internal fixation with NCB Zimmer plates, three fractures were managed with intramedullary device as the implant was compatible with intramedullary device implantation, and three patients had revision arthroplasty due to implant loosening and poor bone stock. Patients were then analyzed with postoperative radiological and clinical evidence of fracture union using the last clinic letter and radiographs from the hospital data and any complication that was developed after the treatment. Patient mobility status before and after the fracture was also reviewed through the patient data.

Data analysis was done using R software version 4.3.3 (R Foundation for Statistical Computing, Vienna, Austria). Descriptive statistics were computed. Comparison of mortality was done among genders, age groups, operation types, fracture locations, complexity of hip fractures, hospitals, and days to operation using the chi-square test for categorical data and the Mann-Whitney U test for numerical data. Logistic regression analysis was conducted with mortality within six months as the dependent variable and the age of the patients and operation status as independent variables to control for confounders. The level of significance was set at p < 0.05.

## Results

The mean time to operate was 3.25 ± 1.11 days. The study involved 100 patients with periprosthetic fractures. The mean age of the patients was 82.22 years (SD: 6.90). There was a slightly higher proportion of males (58 participants) compared to females (42 participants). The majority of fractures were located in the hip (73 participants, 73%), with the remaining 27 participants (27%) having knee fractures. Most patients were treated at DGHs (86 participants, 86%), while 14 participants (14%) were treated at regional orthopedic hospitals (ROHs). Among the 73 patients with hip fractures, the complexity of the fractures was classified according to the Vancouver classification. The distribution was as follows: type A fractures in eight patients (10.96%), type B1 in 29 patients (39.73%), type B2 in 16 patients (21.92%), type B3 in 12 patients (16.44%), and type C in eight patients (10.96%) (Table 1).

**Table 1:** Distribution of age, gender, fracture type, hospital of treatment, and complexity of hip fracture among patients with periprosthetic fractures DGH: district general hospital; ROH: regional orthopedic hospital

Characteristic	N = 100
Age, mean ± SD	82.22 ± 6.90
Gender, n (%)	
Female	42 (42.00)
Male	58 (58.00)
Fracture, n (%)	
Hip	73 (73.00)
Knee	27 (27.00)
Hospital, n (%)	
DGH	86 (86.00)
ROH	14 (14.00)
Complexity of hip based on Vancour classification (n = 73)	
A	8 (10.96)
B1	29 (39.73)
B2	16 (21.92)
B3	12 (16.44)
C	8 (10.96)

**Figure 1:**
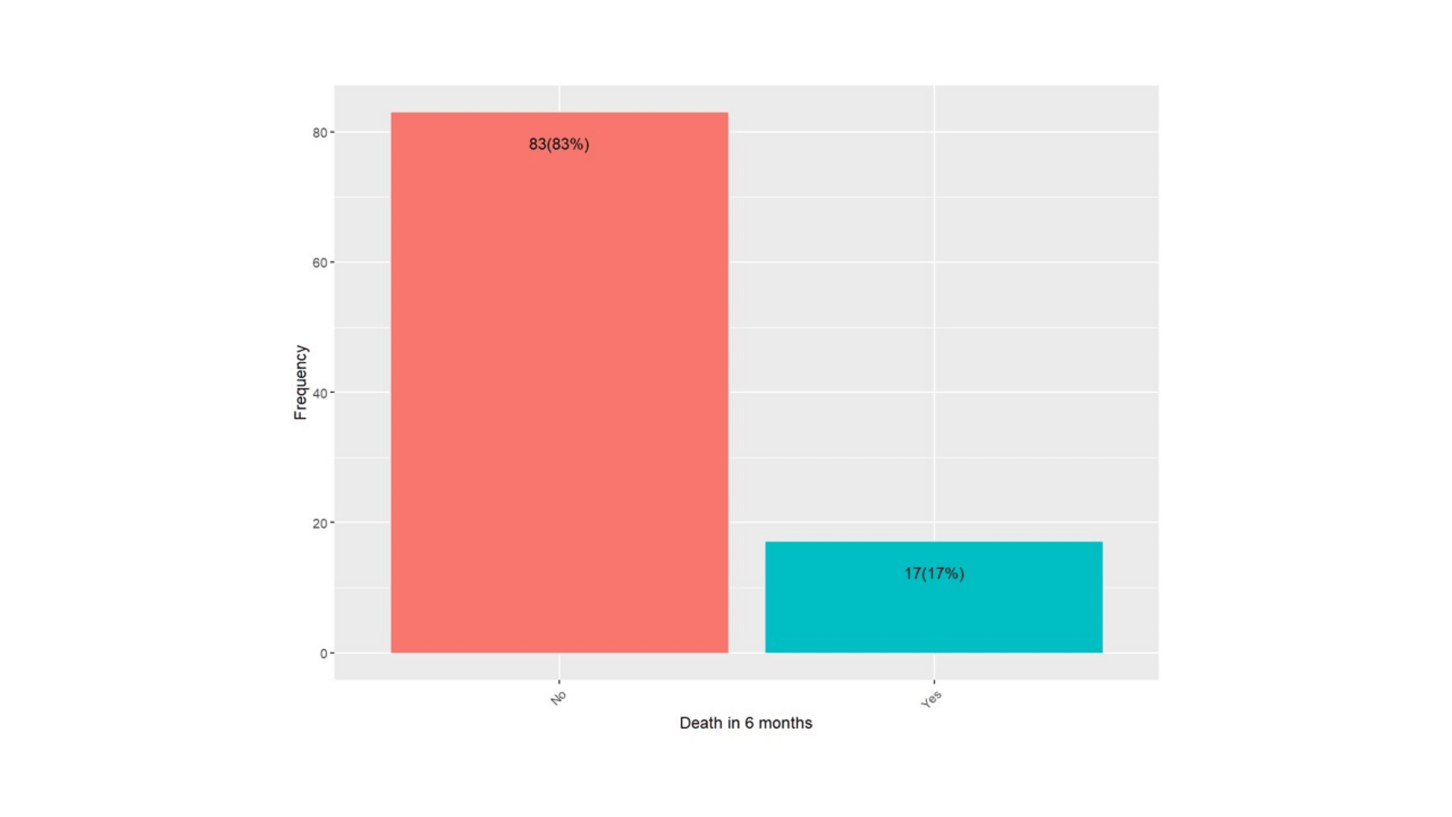
Mortality in six months in patients with periprosthetic fractures

**Figure 2:**
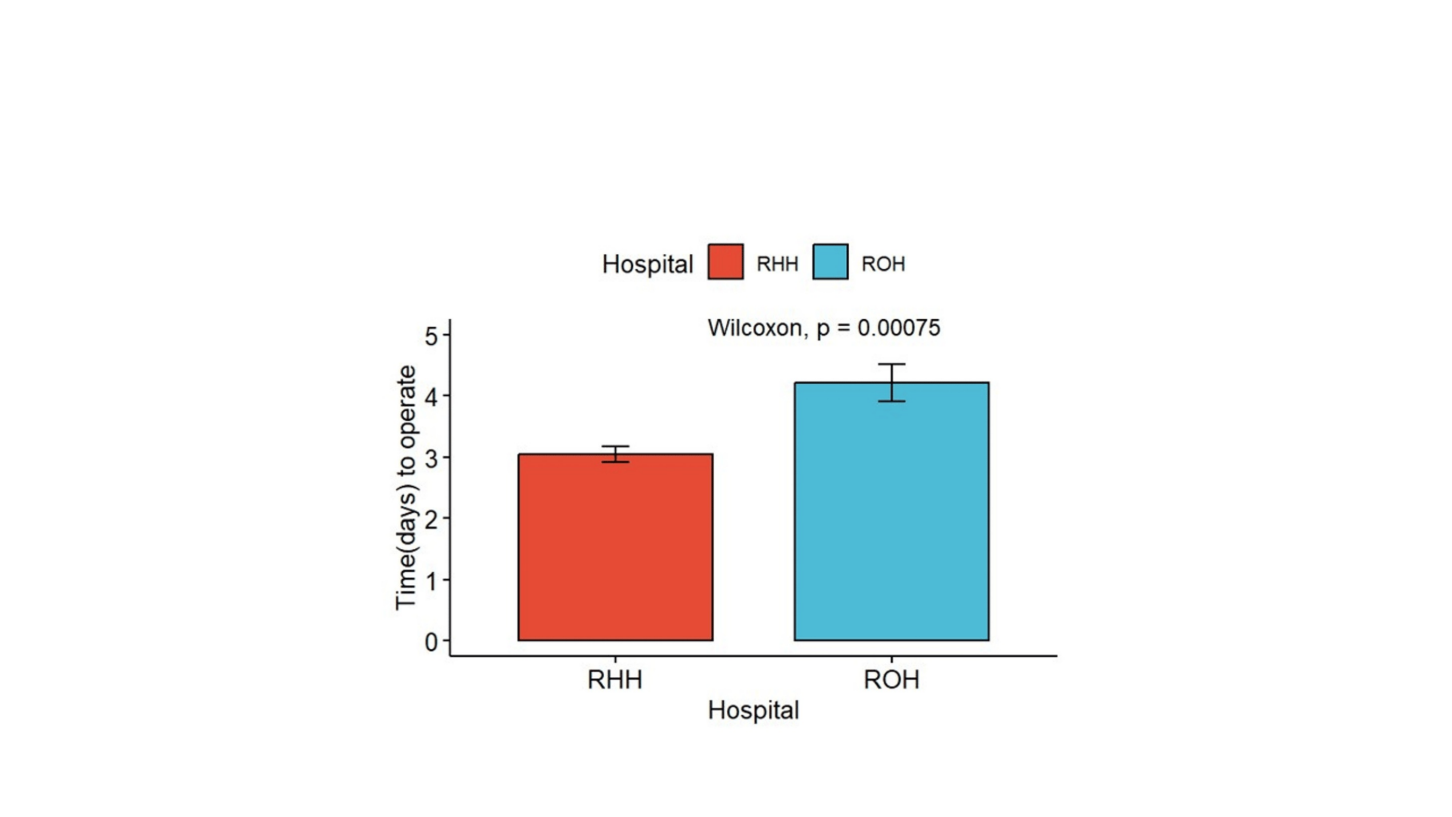
Days to operation in both hospitals RHH: Russells Hall Hospital; ROH: regional orthopedic hospital

Mortality in six months in patients with periprosthetic fractures was 17% (Figure 1). Figure 2 shows that the mean days to operations were higher in ROH than in Russells Hall Hospital (RHH), and the difference was statistically significant (p = 0.00075).

The study assessed mortality within six months among patients with periprosthetic fractures and analyzed various factors, including gender, age, type of operation, fracture location, complexity of hip fracture, hospital of treatment, and days to operation. There was no statistically significant difference in mortality between females (n = 9; 52.94%) and males (n = 8; 47.06%) (p = 0.3). However, a significant difference was found between age groups, with those aged 81 and above having a higher mortality rate (n = 14; 82.35%) compared to those aged 55-80 years (n = 3; 17.65%) (p = 0.04). Mortality was significantly higher in patients who did not undergo surgery (n = 9; 52.94%) compared to those who did (n = 8; 47.06%) (p = 0.003). No significant difference in mortality was observed between hip fractures (n = 12; 70.59%) and knee fractures (n = 5; 29.41%) (p = 0.8). Among patients with hip fractures, mortality did not significantly differ across the complexity classifications (p = 0.5). Specific mortality rates were as follows: type A (n = 3; 17.65%), type B1 (n = 4; 23.53%), type B2 (n = 1; 5.88%), type B3 (n = 2; 11.76%), and type C (n = 2; 11.76%). The majority of deaths occurred in patients treated at DGH (94.12%) compared to ROH (5.88%) (p = 0.5). The mean time to operation was not significantly different between those who died (3.78 ± 0.972 days) and those who survived (3.19 ± 1.12 days) (p = 0.5) (Table 2).

**Table 2:** Comparison of mortality among genders, age groups, operations , fracture location, complexity of hip fracture, hospital, and days to operate DGH: district general hospital; ROH: regional orthopedic hospital

Characteristic	Died in 6 months, N = 17	Alive, N = 83	p-value*
Gender, n(%)			0.3
Female	9 (52.94)	33 (39.76)	
Male	8 (47.06)	50 (60.24)	
Age group (years), n(%)			0.04
81 and above	14 (59.04)	49 (82.35)	
55-80	3 (40.96)	34 (17.65)	
Operative versus nonoperative n(%)			0.003
Nonop	9 (52.94)	14 (16.87)	
Op	8 (47.06)	69 (83.13)	
Fracture location, n(%)			0.8
Hip	12 (70.59)	61 (73.49)	
knee	5 (29.41)	22 (26.51)	
Complexity of hip fracture, n(%)			0.5
A	3 (17.65)	5 (6.02)	
B1	4 (23.53)	25 (30.12)	
B2	1 (5.88)	15 (18.07)	
B3	2 (11.76)	10 (12.05)	
C	2 (11.76)	6 (7.23)	
NA	5 (29.41)	22 (26.51)	
Hospital, n(%)			0.5
DGH	16 (94.12)	70 (84.34)	
ROH	1 (5.88)	13 (15.66)	
Days to operate, , mean± SD	3.78±0.972	3.19±1.12	0.5**

The multivariate analysis explored the association between mortality within six months and predictors such as age and operation status. For age, patients aged 81 and above had a higher mortality rate (22.2%) compared to those aged 55-80 (8.1%). In the univariable analysis, the odds ratio (OR) for mortality in the 55-80 age group was 0.31 (95% CI: 0.07-1.03, p = 0.081), indicating a trend toward lower mortality in this younger age group, although this was not statistically significant. In the multivariable analysis, after adjusting for other factors, the OR for the 55-80 age group was 0.32 (95% CI: 0.07-1.13, p = 0.103), which also did not reach statistical significance. For operation status, patients who did not undergo surgery had a significantly higher mortality rate (39.1%) compared to those who were operated on (10.4%). The univariable analysis showed an OR of 0.18 (95% CI: 0.06-0.55, p = 0.003) for the operated group, indicating a significantly lower risk of mortality compared to the nonoperated group. This association remained significant in the multivariable analysis, with an OR of 0.18 (95% CI: 0.06-0.57, p = 0.004) (Table 3).

**Table 3:** Multivariate analysis for mortality with respect to age and operation

Predictor	Characteristics	Death in 6 months		Univariable	p-value	Multivariable	p-value
		absent	Yes	OR (95% CI)*		OR (95% CI)*	
Age (years)	81 and above	49 (77.8)	14 (22.2)	-		-	
	55-80	34 (91.9)	3 (8.1)	0.31(0.07-1.03)	0.081	0.32 (0.07-1.13)	0.103
Operation	Nonoperated	14 (60.9)	9 (39.1)	-		-	
	Operated	69 (89.6)	8 (10.4)	0.18 (0.06-0.55)	0.003	0.18 (0.06-0.57)	0.004

## Discussion

The study population consisted of 100 patients with periprosthetic fractures, with a mean age of 82.22 ± 6.9 years. The slightly higher proportion of males (58%) compared to females (42%) is consistent with other studies showing a higher incidence of fractures in men. However, the literature indicates that periprosthetic fractures are more common in females (OR = 1.53, p < 0.05) than in males [7]. This discrepancy could be due to differences in study populations, fracture definitions, or other demographic factors not accounted for in this analysis. 

The majority of fractures were located in the hip (73%), aligning with the known prevalence of hip fractures in the elderly due to osteoporosis and falls [1]. Periprosthetic fractures are more common around the hip than the knee due to several factors. The hip joint experiences greater biomechanical stress from weight-bearing activities and has a wider range of motion, increasing fracture risk [8]. Elderly patients, who are more likely to undergo hip replacements, often suffer from osteoporosis and are prone to falls, further elevating the incidence of hip fractures [9]. Additionally, the insertion of the femoral stem during hip replacement creates stress points in the bone, making it more susceptible to fractures [10]. These factors, combined with the high prevalence of hip replacement surgeries, contribute to the higher occurrence of periprosthetic fractures in the hip compared to the knee.

The complexity of hip fractures, classified according to the Vancouver classification, showed a diverse distribution, with the majority being type B1 (39.73%). The distribution of fracture types highlights the varying challenges in managing periprosthetic fractures, with type B fractures often requiring more complex surgical interventions. Similar results were found in a previous study that type B1 was common hip fractures [11].

Most patients were treated at DGH (86%), while a smaller proportion received care at ROH (14%). This disparity might be due to the broader geographic distribution and accessibility of DGHs compared to specialized ROHs, which tend to be fewer and are located in major urban centers.

The overall mortality rate within six months was 17%, reflecting the high-risk nature of periprosthetic fractures in an elderly population. The significant difference in mortality between age groups, with those aged 81 and above having a higher mortality rate (n = 14; 82.35%) compared to those aged 55-80 (n = 3; 17.65%) (p = 0.04), is expected given the increased vulnerability and comorbidities in older patients. Previous literature shows that mortality increases with age among patients with periprosthetic fractures [12]. Another study on the "Morbidity of Periprosthetic Hip and Knee Fractures" investigated mortality rates in patients with periprosthetic fractures. It found that age at the time of surgery was not significantly associated with increased mortality rates in either the univariate or multivariate Cox regression model (p = 0.169), suggesting that age alone did not predict higher mortality in this patient population. Instead, other factors may play a more crucial role in determining outcomes for these patients [13].

Gender did not significantly affect mortality, suggesting that both males and females are equally susceptible to the risks associated with periprosthetic fractures once they occur. Similarly, no significant difference in mortality was observed between hip fractures (n = 12; 70.59%) and knee fractures (n = 5; 29.41%) (p = 0.8), indicating that the location of the fracture may not be as critical a determinant of mortality as other factors.

A key finding was the significantly higher mortality rate in patients who did not undergo surgery (n = 9; 52.94%) compared to those who did (n = 8; 47.06%) (p = 0.003). The multivariate analysis confirmed this association, with an OR of 0.18 (95% CI: 0.06-0.57, p = 0.004) for operated patients, suggesting that surgical intervention substantially reduces the risk of mortality. This underscores the importance of timely and appropriate surgical management in improving survival outcomes for patients with periprosthetic fractures.

The majority of deaths occurred in patients treated at DGHs (n = 16; 94.12%) compared to ROHs (n = 1; 5.88%) (p = 0.5). Although not statistically significant, this finding may indicate differences in the level of care or resources available at these institutions. The mean time to operation was not significantly different between those who died (3.78 ± 0.972 days) and those who survived (3.19 ± 1.12 days) (p = 0.5). However, Figure 2 showed that the mean days to operation were higher in ROHs than DGHs, and this difference was statistically significant (p = 0.00075). Delays in surgery can exacerbate complications and increase mortality risk, highlighting the need for prompt surgical intervention.

The multivariate analysis for mortality with respect to age and operation status further clarified the predictors of mortality. For age, while older patients (81 and above) had a higher mortality rate, the adjusted OR for the 55-80 age group (0.32, 95% CI: 0.07-1.13, p = 0.103) did not reach statistical significance, possibly due to the small sample size or other confounding factors. For operation status, the significant reduction in mortality risk for operated patients (OR: 0.18, 95% CI: 0.06-0.57, p = 0.004) emphasizes the critical role of surgical treatment in improving patient outcomes [14].

A previous study also reported that delaying surgery for patients with periprosthetic fractures beyond two days from admission has been associated with an increased one-year mortality rate, independent of comorbidities [14]. Others also found similar observations in hip fracture patients, highlighting the critical importance of early surgical intervention. Early surgery is recommended for medically stable patients to minimize complications associated with prolonged immobilization, thereby improving outcomes and reducing mortality risks in this vulnerable population [15,16].

Our study also has its own limitations, and the data size can be increased to conclude further detailed results. Another limitation is that we collected data from a single hospital, and a multicenter study can provide further insights. Another limitation is to consider all comorbidities of the patients and then implement them into the results.

## Conclusions

In conclusion, age and operative management were significant factors associated with mortality in patients with periprosthetic fractures. Specifically, older age (81 years and above) and nonoperative management were linked to higher mortality rates. Gender, fracture location, complexity of hip fracture, hospital of treatment, and days to operation did not show significant associations with mortality.

## References

[1] Benkovich V, Klassov Y, Mazilis B, Bloom S (2020). Periprosthetic fractures of the knee: a comprehensive review. Eur J Orthop Surg Traumatol.

[2] Berry DJ (2003). Periprosthetic fractures associated with osteolysis: a problem on the rise. J Arthroplasty.

[3] Solarino G, Vicenti G, Moretti L, Abate A, Spinarelli A, Moretti B (2014). Interprosthetic femoral fractures-a challenge of treatment. A systematic review of the literature. Injury.

[4] Pivec R, Issa K, Kapadia BH, Cherian JJ, Maheshwari AV, Bonutti PM, Mont MA (2015). Incidence and future projections of periprosthetic femoral fracture following primary total hip arthroplasty: an analysis of international registry data. J Long Term Eff Med Implants.

[5] Streubel PN (2013). Mortality after periprosthetic femur fractures. J Knee Surg.

[6] Finlayson G, Tucker A, Black ND, McDonald S, Molloy M, Wilson D (2019). Outcomes and predictors of mortality following periprosthethic proximal femoral fractures. Injury.

[7] Zhu Y, Chen W, Sun T, Zhang X, Liu S, Zhang Y (2015). Risk factors for the periprosthetic fracture after total hip arthroplasty: a systematic review and meta-analysis. Scand J Surg.

[8] Kubiak EN, Beebe MJ, North K, Hitchcock R, Potter MQ (2013). Early weight bearing after lower extremity fractures in adults. J Am Acad Orthop Surg.

[9] Ferrari S, Reginster JY, Brandi ML (2016). Unmet needs and current and future approaches for osteoporotic patients at high risk of hip fracture. Arch Osteoporos.

[10] Savio D, Bagno A (2022). When the total hip replacement fails: a review on the stress-shielding effect. Processes.

[11] Marsland D, Mears SC (2012). A review of periprosthetic femoral fractures associated with total hip arthroplasty. Geriatr Orthop Surg Rehabil.

[12] Märdian S, Perka C, Schaser KD, Gruner J, Scheel F, Schwabe P (2017). Cardiac disease and advanced age increase the mortality risk following surgery for periprosthetic femoral fractures. Bone Joint J.

[13] Smolle MA, Hörlesberger N, Maurer-Ertl W, Puchwein P, Seibert FJ, Leithner A (2021). Periprosthetic fractures of hip and knee-a morbidity and mortality analysis. Injury.

[14] Bhattacharyya T, Chang D, Meigs JB, Estok DM 2nd, Malchau H (2007). Mortality after periprosthetic fracture of the femur. J Bone Joint Surg Am.

[15] Orosz GM, Magaziner J, Hannan EL (2004). Association of timing of surgery for hip fracture and patient outcomes. JAMA.

[16] Crowninshield RD, Rosenberg AG, Sporer SM (2006). Changing demographics of patients with total joint replacement. Clin Orthop Relat Res.

